# Application of a Prediction Error Theory to Pavlovian Conditioning in an Insect

**DOI:** 10.3389/fpsyg.2018.01272

**Published:** 2018-07-23

**Authors:** Makoto Mizunami, Kanta Terao, Beatriz Alvarez

**Affiliations:** ^1^Faculty of Science, Hokkaido University, Sapporo, Japan; ^2^Graduate School of Life Sciences, Hokkaido University, Sapporo, Japan

**Keywords:** blocking, classical conditioning, cricket, dopamine, error-correction learning, invertebrate, octopamine, Rescorla–Wagner model

## Abstract

Elucidation of the conditions in which associative learning occurs is a critical issue in neuroscience and comparative psychology. In Pavlovian conditioning in mammals, it is thought that the discrepancy, or error, between the actual reward and the predicted reward determines whether learning occurs. This theory stems from the finding of Kamin’s blocking effect, in which after pairing of a stimulus with an unconditioned stimulus (US), conditioning of a second stimulus is blocked when the two stimuli are presented in compound and paired with the same US. Whether this theory is applicable to any species of invertebrates, however, has remained unknown. We first showed blocking and one-trial blocking of Pavlovian conditioning in the cricket *Gryllus bimaculatus*, which supported the Rescorla–Wagner model but not attentional theories, the major competitive error-correction learning theories to account for blocking. To match the prediction error theory, a neural circuit model was proposed, and prediction from the model was tested: the results were consistent with the Rescorla–Wagner model but not with the retrieval theory, another competitive theory to account for blocking. The findings suggest that the Rescorla–Wagner model best accounts for Pavlovian conditioning in crickets and that the basic computation rule underlying Pavlovian conditioning in crickets is the same to those suggested in mammals. Moreover, results of pharmacological studies in crickets suggested that octopamine and dopamine mediate prediction error signals in appetitive and aversive conditioning, respectively. This was in contrast to the notion that dopamine mediates appetitive prediction error signals in mammals. The functional significance and evolutionary implications of these findings are discussed.

## Introduction

Pavlovian (or classical) conditioning is a form of associative learning found in many vertebrates and invertebrates (Perry et al., 2013) that is fundamental for animals’ survival since it allows them for finding suitable food, avoiding toxic food, escaping from predators, and detecting mates. This type of learning occurs when an originally unimportant stimulus (conditioned stimulus, CS) becomes associated with a biologically significant stimulus (unconditioned stimulus, US) such that it induces a response (conditioned response, CR) to the CS thereafter. The error-correction learning rule has been thought to account for associative learning in mammals (Pearce, 2008; Mazur, 2013) but little is known about whether the same is true for any species of invertebrates (for earlier attempts in honey bees, see Greggers and Menzel, 1993; Smith, 1997). In this article, we briefly review some basic knowledge of computational rules governing Pavlovian conditioning in both vertebrates and invertebrates and their possible neural substrates, with a special focus on our recent finding that the error correction learning rule seems to best account for Pavlovian conditioning in crickets.

## Prediction Error Theories for Mammalian Pavlovian Conditioning

In associative learning in mammals, a widely accepted view is that the discrepancy, or error, between the reward an animal gets and the reward that the animal predicts (or expects) determines whether learning occurs (Rescorla and Wagner, 1972; Pearce, 2008; Mazur, 2013). The error-correction theory has been applied to learning since at least in 1950s (Bush and Mosteller, 1951) and developed into a refined form in 1970s to account for the finding of blocking phenomenon by Kamin (1969). Blocking takes place when a stimulus (X) that had been paired with a US blocks the subsequent association of a novel stimulus (Y) in a second training phase in which the novel stimulus is presented in compound with X and reinforced by the same US. After this training, when the response to Y alone is tested, it is typically observed that animals do not respond to this stimulus (but notice also that some researchers like, Maes et al., 2016, reported difficulties in replicating blocking effect in rats). The finding of the blocking effect suggests that the strength of temporal contingency (correlation) between the CS and the US, known as a critical factor for conditioning to occur (Rescorla, 1968), is not the only factor that determines the occurrence of learning. Kamin proposed that “surprise” is necessary for learning, and that learning about a stimulus (Y) is blocked when the US is fully predicted by another stimulus (X). This proposition was later formulated into the Rescorla–Wagner model, the most influential form of the error-correction learning theory (Rescorla and Wagner, 1972), which assumes that the discrepancy between the strength of the actual US and total strengths of the predicted US by all the CSs determines the amount of learning (**Table 1A**). Subsequent studies in mammals suggested that dopamine (DA) neurons in the ventral tegmental area of the midbrain mediate prediction error signals for appetitive US, which provided the basis to investigate neural circuit mechanisms of Pavlovian conditioning (Schultz, 2013; Steinberg et al., 2013).

**Table 1:** Error-correction learning theories to account for blocking.

Theory	Equation
**A.** Rescorla-Wagner model (Rescorla and Wagner, 1972)	ΔV = α(λ–V_Σ_)
**B.** Attentional theory by Mackintosh (1975)	ΔV = α_A_(λ–V_A_)
	α_A_ is positive if | λ–V_A_| < | λ–V_X_|
	α_A_ is negative if | λ–V_A_|≥| λ–V_X_|
**C.** Attentional theory by Pearce and Hall (1980)	ΔV_A_ = S_A_α_A_λ
	α_A_^n^ = |λ^n−1^–V_Σ_^n−1^|

*In **A**, V is associative strength that refers to the strength of the CS-US, which corresponds to US prediction, ΔV is the change in V that results from a particular conditioning trial, V_Σ_ is total association strengths of all CSs present in a conditioning trial, λ is the magnitude of the US and reflects the maximum strength of the CS-US association that can be achieved, and α is a learning-rate parameter reflecting the intensity of the CS. The model accounts for blocking by decreased (λ–V_Σ_) reflecting a change of V as a result of preceding conditioning trials. In **B**, α_A_ is the amount of attention to CS_A_, V_X_ is the associative strength of all stimuli other than CS_A_ present in a given trial. The theory accounts for blocking by decreased α_A_ as a result of preceding trials. In **C**, α_A_^n^ is the amount of attention to CS_A_ of the n-th trial, and S_A_ is a parameter that depends on intensity of CS_A_. The model accounts for blocking by decreased α_A_. Description of equations follows Pearce and Hall (1980).*

There are theories other than the Rescorla–Wager model that can account for the blocking effect (Miller et al., 1995; Pearce, 2008; Mazur, 2013). The most influential competitive ones are the attentional theories proposed by Mackintosh (1975) and by Pearce and Hall (1980), which are refined versions of the error-correction learning theory and account for blocking by decreased attention to the CS (**Tables 1B,C**). It can be stated that Rescorla–Wagner model focuses on US processing whereas attentional models focus more on CS processing. Another notable theory is the comparator hypothesis (Miller and Matzel, 1988), which accounts for blocking by competition between CSs during the memory retrieval process. Remarkably, although efforts have been directed to experimentally test these different theories, which of the theories mentioned best accounts for computational rules governing Pavlovian conditioning remains unclear in any conditioning system (Miller et al., 1995; Pearce, 2008; Mazur, 2013).

## Studies on Neural Processing Underlying Pavlovian Conditioning in Invertebrates

Whether error-correction learning models such as the Rescorla–Wagner model represent computational rules underlying learning in any species of invertebrates remained unknown until recently. One of the reasons for the lack of such study is the difficulty in establishing experimental procedures to convincingly demonstrate blocking. In insects, for example, some earlier studies in honey bees (e.g., Smith, 1997; Hosler and Smith, 2000) showed a blocking-like effect but more recent studies failed to establish blocking as a robust phenomenon in honey bees (Guerrieri et al., 2005; Blaser et al., 2006, 2008). Second, although blocking has been reported in the slug *Limax maximus* (Sahley et al., 1981), the snail *Cornu aspersum* (formerly *Helix aspersa*, Acebes et al., 2009; Prados et al., 2013a) and the planaria *Dugesia tigrina* (Prados et al., 2013b) no attempts have been made to investigate which computational model best accounts for blocking in any of these invertebrate species.

Many of the previous studies on the neural basis of Pavlovian conditioning in invertebrates focused on clarifying the cellular and molecular mechanisms that allow animals to detect the coincident and correlated occurrence of the CS and the US, a pre-requisite for Pavlovian conditioning. In Pavlovian conditioning of gill withdrawal responses in the sea hare *Aplysia californica*, it has been demonstrated that neural signals mediating CS and US converge in some neurons of the nervous system and that type 1 adenylyl cyclase (AC), which catalyzes ATP to produce cAMP, and the *N*-methyl-*D*-aspartate (NMDA) receptor, a type of glutamate receptor, serve as key molecules for the detection of coincident arrival of CS and US signals to these neurons to lead to modification of the efficacy of synaptic transmission that underlies conditioning (Abrams and Kandel, 1988; Hawkins and Byrne, 2015). Similarly, in the fruit-fly *Drosophila melanogaster*, it has been shown that type 1 AC in intrinsic neurons (Kenyon cells) of the mushroom body, a higher-order associative center in the insect brain (Menzel and Giurfa, 2006; Watanabe et al., 2011; Burke et al., 2012; Liu et al., 2012), serve as key molecules to detect coincident arrival of the olfactory CS and the electric shock or the sucrose US signals to these neurons for achieving conditioning (Davis, 2005; Gervasi et al., 2010). However, whether such coincidence detection mechanisms are sufficient to achieve Pavlovian conditioning in these species remains unclear.

## Neural Substrates Underlying Pavlovian Conditioning in Crickets

We recently investigated whether blocking occurs in Pavlovian conditioning in the cricket *Gryllus bimaculatus*. Crickets are newly emerging experimental animals in which associative learning is explored by pairing visual or olfactory cues with either water (to elicit appetitive learning) or with sodium chloride (to induce aversive learning). With these procedures, the neural mechanisms that are involved in both the acquisition and the retrieval of the CR of Pavlovian conditioning have been investigated in some detail (Matsumoto and Mizunami, 2002; Matsumoto et al., 2006, 2018; Mizunami et al., 2014, 2015; Matsumoto Y. et al., 2016). For example, concerning the acquisition of both olfactory and visual learning, we showed that pharmacological blockade of octopamine (OA)-ergic synaptic transmission impairs appetitive but not aversive Pavlovian conditioning, whereas pharmacological blockade of DA-ergic transmission impairs aversive conditioning but not appetitive conditioning (Unoki et al., 2005, 2006; Mizunami et al., 2009; Nakatani et al., 2009; Matsumoto et al., 2015; Mizunami and Matsumoto, 2017). The results obtained in the pharmacological studies were further confirmed in subsequent studies on the effects of knockout or knockdown of genes that code DA receptors or OA receptors by the CRISPR/cas9 system (Awata et al., 2015) or by RNAi (Awata et al., 2016). These findings suggest that OA neurons and DA neurons mediate neural signals representing appetitive and aversive US, respectively, in both olfactory and visual conditioning. Moreover, OA and DA neurons are also involved in the execution of the CR (or in the retrieval of the memory): blockade of OA-ergic transmission impaired CR execution after appetitive conditioning, but not after aversive conditioning with sodium chloride, and blockade of DA-ergic transmission impaired the execution of the CR after aversive conditioning but not after appetitive conditioning (Mizunami et al., 2009). Therefore, it has been concluded that activation of OA neurons is needed for the execution of a CR after appetitive conditioning, whereas activation of DA neurons is needed for the execution of an aversive CR. These results have been integrated in a neural circuit model for Pavlovian conditioning in crickets, which is assumed to represent neural circuitry of the mushroom body (Mizunami et al., 2009). The model accounted for two higher-order learning phenomena, namely second-order conditioning (Mizunami et al., 2009) and sensory preconditioning (Matsumoto et al., 2013). This model provided the basis to construct a model to account for blocking described in subsequent sections.

Roles of OA and DA in mediating appetitive and aversive signals in Pavlovian learning have also been reported in honey bees (Hammer and Menzel, 1998; Farooqui et al., 2003; Vergoz et al., 2007, but see Perry et al., 2016 for bumblebees). In fruit-flies, on the other hand, it has been concluded that different classes of dopamine neurons projecting to the mushroom body mediate appetite and aversive signals (Burke et al., 2012; Liu et al., 2012). It seems that the neurotransmitter mediating appetitive signals differs in different species of insects, although that mediating aversive signals is conserved among insects.

## Applicability of Prediction Error Theory to Pavlovian Conditioning in Crickets

Experiments showing blocking with crickets were conducted, at first, with an appetitive procedure in which water was used as the US. Crickets were subjected to four conditioning trials in which they were exposed to stimulus X immediately before the presentation of water (X+) and were then subjected to compound trials in which stimulus X was presented together with a new stimulus Y followed by the same US (XY+), X and Y being stimuli of different sensory modalities (an olfactory and a visual pattern stimulus, counterbalanced; Terao et al., 2015). Crickets subjected to this training did not respond to Y. In contrast, control crickets that were exposed to unpaired presentations of X and the US (X/+) and then to paired and reinforced presentations of the compound (XY+) or crickets that received only XY+ training exhibited normal learning of Y. Similar results were also obtained in experiments in which blocking was assessed by means of an aversive conditioning procedure (i.e., NaCl was used as the US; Terao and Mizunami, 2017). The results showed that blocking occurs in both appetitive conditioning and aversive conditioning in crickets.

As already mentioned, the most influential models to account for blocking are the Rescorla-Wagner model (Rescorla and Wagner, 1972), the attentional theories proposed by Mackintosh (1975) and by Pearce and Hall (1980), and the retrieval theory (or comparator hypothesis) proposed by Miller and Matzel (1988). However, whether blocking is better accounted for by any of the mentioned models has not been tested in an invertebrate species, except that Smith (1997) examined blocking in honey bees and argued that the Rescorla–Wagner model can at least in part account for blocking but the attentional theories seem not to account for it. To discriminate among these models, one-trial appetitive blocking experiments were performed. In such experiments crickets received X+ training trials followed by one single XY+ training trial. We used one compound conditioning trial because the Rescorla–Wagner model predicts that such training will result in blocking of Y, whereas attentional theories do not (Mackintosh, 1975; Pearce and Hall, 1980). Our results showed that crickets that received X+ training followed by one XY+ compound-conditioning trial did not respond to Y. In contrast, control crickets that were exposed to unpaired presentations of X and the US followed by one XY+ compound training trial or that received only one XY+ training trial exhibited normal learning of Y. The results supported the Rescorla–Wagner model but not the attentional theories for appetitive conditioning (Terao et al., 2015). We also investigated whether blocking with one XY+ training trial can be accounted for by assuming simple selective attentional process not coupled to error-correction learning, and the results were not consistent with this possibility (Terao et al., 2015). In the case of aversive conditioning (i.e., using NaCl as the US), however, a blocking experiment with one compound trial could not be performed since previous studies have shown that one aversive X+ conditioning trial does not result in aversive learning (Unoki et al., 2005, 2006). Therefore, discrimination of the Rescorla–Wagner model and attentional theories in aversive conditioning remains to be explored. The possible applicability of the retrieval theory will be discussed in a later section.

**FIGURE 1:**
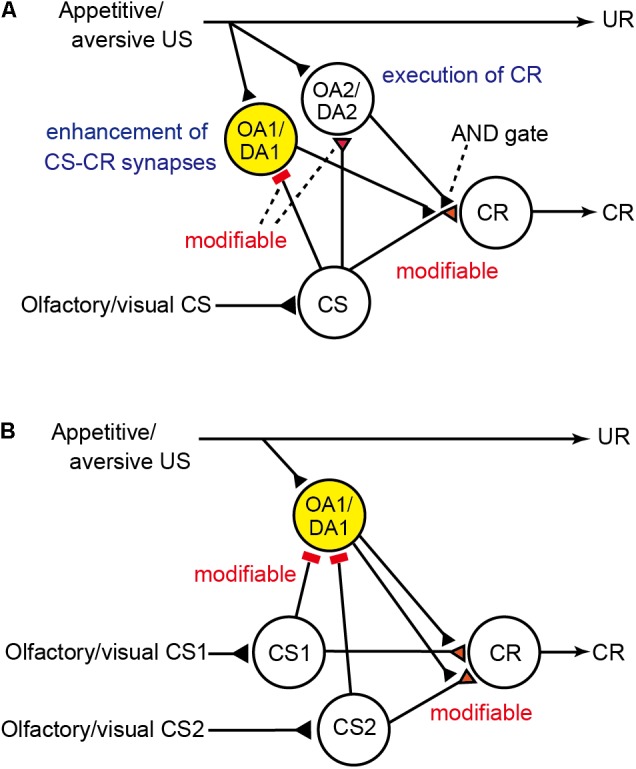
Neural models of Pavlovian conditioning in crickets proposed by Terao et al. (2015) and Terao and Mizunami (2017). **(A)** Description of the model that has been revised from the model by Mizunami et al. (2009) to match the prediction error theory. The model assumes two classes of OA and DA neurons. One is “OA1/DA1” neurons (colored in yellow) that govern enhancement of “CS-CR” synapses (but not execution of a CR). The other is “OA2/DA2” neurons that govern execution of a CR or memory retrieval (but not enhancement of “CS-CR” synapses). The model also assumes that (1) “CS” neurons [which may represent intrinsic neurons (Kenyon cells) of the mushroom body] that convey signals for CS make silent or weak synaptic connections with dendrites of “CR” neurons [which may represent efferent (output) neurons of the lobes (output regions) of the mushroom body], activation of which leads to a CR, but these synaptic connections are silent or very weak before conditioning, (2) “OA1/DA1” neurons receive excitatory synapses that represent appetitive/aversive US signals and silent or very weak inhibitory synapses from “CS” neurons before training, which are strengthened by CS-US pairing, (3) during training, “OA1/DA1” neurons receive excitatory synaptic input that represents actual US and inhibitory input from “CS” neurons that represents US prediction by CS, and thus their activities represent US prediction error signals, (4) “OA2/DA2” neurons receive excitatory synapses that represent US signals and silent or very weak excitatory synapses from “CS” neurons before training, which are strengthened by CS-US pairing, and (5) “OA2/DA2” neurons make synaptic connections with axon terminals of “CS” neurons, and coincident activation of “CS” neurons and “OA2/DA2” neurons is needed for activation of “CR” neurons (AND gate) and for production of a conditioned response. Presentation of a CS after CS-US pairing activates “CS” neurons and then “OA2/DA2” neurons and thus activates “CR” neurons to lead to a CR. Synapses for which the efficacy can be changed by conditioning are colored in red and marked as “modifiable.” Excitatory synapses are marked as triangles, and inhibitory synapses are marked as bars. UR: unconditioned response. **(B)** Accounts for blocking by the model. “OA2/DA2” neurons in the model in **(A)** are not shown in **(B)** for simplicity. The models are modified from Terao et al. (2015) and Terao and Mizunami (2017) with permission.

To account for these findings, we proposed a neural circuit model of Pavlovian conditioning in crickets that matches the Rescorla–Wagner theory (**Figure 1A**; Terao et al., 2015; Terao and Mizunami, 2017), by revising our previous model (Mizunami et al., 2009). The major assumption in our model is that pairing of the CS and the US lead to the enhancement of synaptic transmission from “CS” neurons to three classes of neurons, i.e., “CR,” “OA1/DA1,” and “OA2/DA2” neurons, in which “CS” neurons are neurons mediating signals about CS (which may represent intrinsic neurons of the mushroom body) and “CR” are neurons that lead to the CR when they are activated (which may represent output neurons of the mushroom body lobes). “OA1/DA1” or “OA2/DA2” neurons are separate classes of OA or DA neurons that receive signals about appetitive or aversive USs (which may represent OA or DA neurons projecting to the mushroom body lobes). “OA1/DA1” neurons (colored in yellow in **Figure 1A**) govern enhancement of “CS-CR” synapses (but not execution of a CR) whereas “OA2/DA2” neurons govern execution of a CR (but not enhancement of “CS-CR” synapses) and here we focus on the former neurons. The model assumes that “OA1/DA1” neurons are critical for error-correction computation, in that (1) the efficacy of “CS-OA1/DA1” inhibitory synapses increases by coincident activation of “CS” and “OA1/DA1” neurons during CS-US pairing trials, (2) inhibitory inputs to “OA1/DA1” neurons represent signals about US prediction by the CS whereas excitatory inputs to these neurons represent US signals, (3) responses of “OA1/DA1” neurons during CS-US pairing trials, hence, represent US prediction error signals, and (4) after sufficient amount of training, responses of “OA1/DA1” neurons during CS-US pairing decrease to the zero level and hence no further enhancement of “CS-CR” synapses occurs. Details of the model are shown in the legend of **Figure 1A**, and how responses of “OA1/DA1” neurons to paired CS-US presentations represent US prediction error signals is described in **Table 2**. As for models of the mushroom body that are intended to account for some other memory tasks, see literatures such as Peng and Chittka (2017) and Roper et al. (2017).

**Table 2:** Information coded in the responses of “OA1/DA1” neurons in the model of **Figure 1**.

Stimulus	Before training	After training
US	1 (US)	1 (US)
CS	0	0 [-1 (-USP)]^∗^
CS + US	1 (US)	0 (USPE)

*Responses of “OA1” or “DA1” neurons in the model shown in **Figure 1** to appetitive or aversive US, CS, and paired presentation of CS and US before and after conditioning. These neurons are assumed to govern enhancement of synaptic transmission underlying conditioning. After completion of training, these neurons receive excitatory synaptic input when a US is presented and receive inhibitory synaptic input when a CS is presented, the former representing US signals and the later representing US prediction signals. Paired presentations of CS and US induces no responses to these neurons if US-induced excitatory input was canceled by an inhibitory input induced by a CS. In such situations, no further enhancement of synaptic transmission occurs. USP, US prediction; USPE, US prediction error. Responses are indicated as all or none (1 or 0). ^∗^Negative value in the parentheses indicates inhibitory synaptic input. Based on Terao et al. (2015).*

**Figure 1B** depicts how the model accounts for blocking. CS1-US pairing trials strengthen “CS1-OA1/DA1” inhibitory synapses so that responses of “OA1/DA1” neurons during trials are diminished to the zero level. Therefore, when the CS1-CS2 compound is subsequently presented and reinforced with the same US, “OA1/DA1” neurons produce no responses and hence, no enhancement of “CS2-CR” synapses occur (Terao et al., 2015).

One of the predictions that can be made from the model is that, in the case of appetitive conditioning, blockade of output synapses from OA neurons by administration of an OA receptor antagonist (e.g., epinastine) during the conditioning of a stimulus Y (Y+ training) impairs learning of Y since normal synaptic outputs from “OA1” neurons are needed for enhancement of “CS-CR” synapses. This treatment, however, would not affect the prediction error computation, since synaptic outputs from “OA1” neurons do not participate in prediction error computation (**Figure 1B**; Terao et al., 2015). Therefore, administration of epinastine before Y+ training would still allow for error correction to take place in each trial, even though it prevents an enhancement of “CS-CR” synapses necessary for learning. The model thus predicts that subsequent Y+ training after recovery from the effect of epinastine should produce no learning if the associative strength of the “CS-OA1” synapses reaches the maximum after initial Y+ training. Crickets of the experimental group indeed exhibited no learning of Y. In contrast, crickets in the control group that were administrated with epinastine before unpaired presentation of Y and US and then subjected to Y+ training after recovery from the effect of epinastine exhibited normal learning of Y. We referred to this inhibitory phenomenon as “auto-blocking,” because learning of Y seems to be blocked by the prediction of the US by Y itself (and not by another stimulus, X, as in the case of blocking experiment) (Terao et al., 2015). The absence of CR in the test could also be explained by the comparator model if memory is formed in the second training but not retrieved in the test due to competition of memories formed in the initial and second trainings. Such competition, however, is difficult to assume since results of all our previous studies suggest that no memory is formed in the first training (e.g., Unoki et al., 2005). Taken together, one-trial blocking and the auto-blocking phenomenon suggest that the Rescorla–Wagner model is the one that best accounts for appetitive conditioning in crickets (Terao et al., 2015). In addition, auto-blocking experiments suggest that OA neurons mediate appetitive prediction error signals.

Subsequent studies also showed auto-blocking in an aversive conditioning experiment. Crickets were first administered with a DA receptor antagonist (flupentixol) before training with Y+ (or before exposure to unpaired presentations of Y and + in the case of the control group). As in the previous case, subsequent Y+ training after animals had recovered from the effect of flupentixol did not result in learning of Y (Terao and Mizunami, 2017), whereas animals in the control group showed an increased aversion to Y. The results suggest that the Rescorla–Wagner model or other forms of error-correction learning theories, but not the retrieval theory, best account for aversive conditioning. The results of auto-blocking experiments also suggest that DA neurons mediate aversive prediction error signals.

It should be noted, however, that we do not suggest that error-correction learning theories account for all aspects of Pavlovian conditioning in crickets. The model proposed to account for Pavlovian conditioning in crickets assumes synaptic plasticity in three different synapses in the circuitry and suggests that the plasticity of one type of synapses (“CS-CR” synapses) is governed by US prediction error whereas the plasticity of the other two synapses (“CS-OA1/DA1” and “CS-OA2/DA2” synapses) is governed by coincident occurrence of CS and US. Moreover, we have observed second-order conditioning (Mizunami et al., 2009) in crickets, which is difficult to be accounted for by the Rescorla–Wagner model without appropriate revisions (Miller et al., 1995). We have proposed that these learning phenomena in crickets can be accounted for by neural models that assume no error-correction computation (specifically, by neural pathways involving “OA2/DA2” neurons) (Mizunami et al., 2009; Matsumoto et al., 2013; Terao et al., 2015).

It can be pointed out that major predictions from our model differ from those of the temporal difference (TD) model (Sutton and Barto, 1987), a variant of error-correction learning models and frequently used for simulations of activities of dopamine neurons in the midbrain in primates. It has been shown that those neurons in primates are activated by learned CS and less by predicted US after Pavlovian conditioning, in accordance with the TD model (Schultz, 2015). Interestingly, some of these features have also been found in a ventral unpaired neuron, an OA neuron in the subesophageal ganglion in honey bees that mediates sucrose signals in appetitive olfactory conditioning (Hammer, 1993). In our model, on the other hand, activities representing the US prediction by the CS (i.e., responses to learned CS) and those representing US prediction error (i.e., less responding to predicted US during paired CS-US presentation after training) are assumed in separate classes of aminergic neurons (i.e., “OA2/DA2” and “OA1/OA1” neurons) for simplification of the model. Physiological investigations are needed to clarify the validity of our model.

## Functional and Evolutionary Considerations

The finding that an error-correction learning rule accounts for Pavlovian conditioning in crickets is remarkable since it suggests that the basic computational rules underlying Pavlovian learning in crickets are the same to those in mammals. Error-correction computation, one of fundamental neural computations executed in the mammalian brain, can also be achieved in the small brain of crickets. It is thus of interest to elucidate the neural circuit mechanisms underlying the error-correction learning in crickets, and in other species of invertebrates, to compare them with those in mammals. In mammals, midbrain DA neurons are thought to mediate prediction error signals for appetitive stimuli, and whether DA neurons also mediate aversive prediction error signals is under debate (Schultz, 2013; Matsumoto H. et al., 2016). In mice, it has been suggested that prediction error signals observed in midbrain DA neurons are the result of summation of information across multiple brain areas, rather than prediction error signals being computed in a specific brain area (Tian et al., 2016). In crickets, we hypothesize that OA and DA neurons projecting to the mushroom body mediate appetitive and aversive prediction error signals, respectively (Terao et al., 2015; Terao and Mizunami, 2017). Anatomical and physiological characterizations of these OA and DA neurons should pave the way for elucidating the ubiquity and differences of the neural mechanisms underlying prediction error computation among animals of different phyla.

Some questions arise concerning the functional significance and evolution of the error-correction learning rule underlying Pavlovian conditioning in crickets. An important question is what are the functional advantages of having such associative learning systems in which coincident and correlated occurrence of a CS and a US is not sufficient to lead to learning. To facilitate discussion on this issue, we assume that many of the Pavlovian conditioning systems in invertebrates are based on a simpler learning rule, namely, they are based solely on the detection of coincident or contingent occurrence of a CS and a US, as has been assumed by many neurobiologists. It can be argued that an error-correction learning system is advantageous when multiple CSs occur in association with a US, since, in such a system, the magnitude of learning of a given CS is determined by its relative “surprisingness” or by to what extent the CS predicts the US. This learning system is more efficient in that it prevents learning of redundant cues compared to a learning system that is solely based on the detection of temporal coincidence or contingence, in which all CSs that occur in the same temporal relationship with a US should be equally learned. An error-correction learning, however, should have a cost, in that it requires elaborate neural circuits in the brain, and the development and maintenance of such circuits should be costly. Such a cost, however, is likely to be moderate since it is affordable for crickets that have only small brains.

Another question to be addressed in the future is to what extent the Pavlovian conditioning system with the error-correction rule is ubiquitous among invertebrates. The blocking phenomenon, a hallmark for the existence of the error-correction learning rule, has so far been reported only in slugs (Sahley et al., 1981), snails (Acebes et al., 2009; Prados et al., 2013a), and planarians (Prados et al., 2013b) but whether it occurs by error-correction learning or by other process, such as cue competition during memory retrieval (Miller and Matzel, 1988) or simple selective attentional process not coupled to error-correction learning (see Terao et al., 2015) has not been investigated. Slugs and snails possess well-developed central nervous systems (Sahley et al., 1981; Loy et al., 2006), comparable to those of insects, and it would be therefore likely that the blocking effect is based on error-correction learning rules as well. On the other hand, since the central nervous system of planarians is much less organized than that of insects, it would be likely that blocking in planarians reflects processes other than error-correction learning. In insects, it is of interest to see whether blocking is based on an error-correction rule in species other than crickets. However, unambiguous evidence of blocking phenomenon has not been found in honey bees (Guerrieri et al., 2005; Blaser et al., 2006, 2008) or in the fruit fly *Drosophila melanogaster* (Young et al., 2011). In the case of honey bees, for example, contradictory results have been reported in the literature from blocking of the CR (Smith, 1997; Hosler and Smith, 2000) to the absence of blocking (Blaser et al., 2006, 2008). Guerrieri et al. (2005) reported blocking, no blocking or even enhanced responding to the blocked element (i.e., augmentation) depending on the odor pairs used in the blocking experiment in honey bees. The reasons for the contradictory results in honey bees remain to be explored.

Finally, phenomena that are not consistent with the Rescorla–Wagner model, such as recovery from extinction, and phenomena that are difficult to be accounted for by the Rescorla–Wagner model without appropriate revisions, such as second-order conditioning, have been reported in some invertebrate species (e.g., Sahley et al., 1981; Loy et al., 2006; Hussaini et al., 2007; Tabone and de Belle, 2011; Alvarez et al., 2014). What neural circuit mechanisms underlie associative learning in these species remains for future subjects.

## Author Contributions

MM, KT, and BA wrote the manuscript and approved the final version.

## Conflict of Interest Statement

The authors declare that the research was conducted in the absence of any commercial or financial relationships that could be construed as a potential conflict of interest.
